# Factors Associated with Suicide Risk Among the Elderly Living Alone in Jeju, Korea

**DOI:** 10.1192/bjo.2026.11080

**Published:** 2026-06-30

**Authors:** Moon-Doo Kim, Won-Myong Bahk, Bo-Hyun Yoon, Kwanghun Lee

**Affiliations:** 1 Jeju National University Hospital, Jeju, Korea, Republic of; 2 Woo and Bhak Psychiatric Clinic, Seoul, Korea, Republic of; 3 Naju National Hospital, Naju, Korea, Republic of; 4 Lee Kwanghun Psychiatric Clinic, Gyeongju, Korea, Republic of

## Abstract

**Aims:**

The growing number of elderly individuals living alone presents a major social issue, encompassing loneliness, isolation, economic and health challenges, and increased risk ofsuicide. This study aimed to identify factors associated with suicide risk among elderly individuals living alone in Jeju.

**Methods:**

A total of 4,742 participants completed questionnaires to assess their sociodemographic characteristics. Depressive symptoms were evaluated using the Short Form Geriatric Depression Scale-Korean version (sGDS-K), with a cutoff score of eight indicating the presence of depressive symptoms. Suicide risk was assessed using the Mini-International Neuropsychiatric Interview-Plus. Multivariate logistic regression analysis was performed to identify significant correlates of suicide risk.

**Results:**

Multivariate logistic regression analysis identified the following factors as significantly associated with suicide risk: poor subjective health status (odds ratio [OR]=1.590, 95% confidence interval [CI]: 1.137–2.224), current drinking (OR=1.511, 95% CI: 1.119–2.042), hypertension (OR=1.419, 95% CI: 1.133–1.778), and sGDS scores ≥8 (OR=4.318, 95% CI: 3.408–5.469).

**Conclusion:**

This study highlights the importance of intensive mental health services and socioeconomic support in preventing suicide among elderly individuals living alone. Targeted interventions should focus on those who have poor subjective health status, are current drinkers, have hypertension, or exhibit depressive symptoms.

